# Magnetic Resonance Cartography of Renal Tubule Volume Fraction During Diuretic Intervention

**DOI:** 10.1111/apha.70095

**Published:** 2025-08-22

**Authors:** Ehsan Tasbihi, Thomas Gladytz, Jason M. Millward, Kathleen Cantow, Erdmann Seeliger, Thoralf Niendorf

**Affiliations:** ^1^ Max‐Delbrück‐Center for Molecular Medicine in the Helmholtz Association (MDC) Berlin Ultrahigh Field Facility (B.U.F.F.) Berlin Germany; ^2^ Charité—Universitätsmedizin Berlin Berlin Germany; ^3^ Experimental and Clinical Research Center A Joint Cooperation Between the Charité Medical Faculty and the Max Delbrück Center for Molecular Medicine Berlin Germany; ^4^ Institute of Translational Physiology Charité—Universitätsmedizin Berlin Berlin Germany

**Keywords:** furosemide, kidney, MRI, tubule system, tubule volume fraction

## Abstract

**Aim:**

The renal tubular volume fraction (TVF) fluctuates under physiological conditions, and is altered in several renal diseases. Tools that enable noninvasive assessment of TVF are currently lacking. Magnetic Resonance (MR) TVF cartography is a novel approach for unraveling renal (patho‐)physiology. Here, we employ MR‐TVF cartography to monitor changes in response to the diuretic furosemide, and examine its role for the interpretation of renal oxygenation assessed by mapping the MRI relaxation time *T*_2_*. We hypothesize that furosemide increases TVF.

**Methods:**

In anesthetized rats (*n* = 7) the MRI relaxation times *T*_2_, *T*_2_*, *T*_2_′ and kidney size were obtained before/following an i.v. bolus of furosemide using a 9.4 Tesla MRI scanner. Spectral analysis of the *T*_2_ signal decay was performed to estimate the number of *T*_2_ components in renal tissue. TVF cartographies were calculated using voxel‐wise bi‐exponential fit of the *T*_2_ decay. Near Infrared Spectroscopy (NIRS, *n* = 9) was used to assess the total hemoglobin concentration (HbT) as a surrogate of renal blood volume.

**Results:**

Furosemide induced changes in renal MRI and NIRS parameters relative to baseline: TVF_CORTEX_ = 31.1%, TVF_OUTER_MEDULLA_ = 30.7%, *T*_2_CORTEX_ = 13.0% and *T*_2_OUTER_MEDULLA_ = 20.6%. HbT_CORTEX_ was reduced by 2.7%. HbT_MEDULLA_ declined by 8.6%. Kidney size showed a modest increase of 2.9%. *T*_2_*_OUTER_MEDULLA_ and *T*_2_ **´**_OUTER_MEDULLA_ rose by 20.5% and 20.2%. *T*_2_*_CORTEX_ and *T*_2_ **´**_CORTEX_ remained unchanged. *T*_2_* and TVF were strongly correlated in the outer medulla and moderately in the cortex.

**Conclusion:**

MR‐TVF cartography is highly relevant for elucidating mechanisms of renal (patho‐)physiology, including the role of renal oxygenation assessed by MRI mapping of renal *T*_2_*.

## 1 Introduction

The fluid volume within the renal tubules fluctuates significantly under physiological conditions and in various clinical scenarios and renal diseases. However, there is currently no available noninvasive method to quantify or investigate the tubular fluid volume and its proportion to the total kidney volume—that is, the tubular volume fraction (TVF). Such a method is urgently needed, since the existing clinical diagnostic tools cannot detect changes in the TVF. The TVF is supposed to be altered in response to changes in the glomerular filtration rate (GFR), tubular water reabsorption, the transmural pressure of the tubules, outflow of urine into the extrarenal urinary tract, and from rarefaction of the tubules.

Changes in GFR are expected to result in parallel changes in TVF. Pathophysiologically relevant decreases in GFR are caused by reduced effective filtration pressure or reduced hydraulic conductance of the glomerular filter in a variety of clinical scenarios [1, 2, 3, 4, 5, 6]. Increases in GFR are typical for the early stages of diabetic kidney disease and for the remaining kidney following unilateral nephrectomy [7, 8].

Alterations in tubular water reabsorption will result in opposite changes of the TVF. Hyperglycaemia leads to reduced water reabsorption; this also happens upon administration of diuretics, particularly osmotic and loop diuretics [9, 10, 11]. Loop diuretics like furosemide are widely used in clinical practice to enhance urine flow rate and NaCl excretion. They inhibit the NKCC2 transporter in the thick ascending limb of the loop of Henle, which results in decreased water reabsorption in the nephron portions distal to the thick ascending limb [11]. Polycystic kidney disease is also characterized by a progressive increase in the TVF [12]. Administration of X‐ray contrast media during transcutaneous cardiac procedures increases tubular fluid viscosity, thereby reducing tubular fluid flow and increasing intratubular pressure, which results in circular distension of the tubules [13]. Conversely, intrarenal edema formation following acute events such as ischemia/reperfusion injury increases intrarenal pressure and may lead to compression of the tubules, since the renal capsule is relatively rigid [14, 15].

Obstructions anywhere in the extrarenal urinary tract that reduce urine outflow will lead to increased TVF. Such obstructions can occur during endourologic procedures and can be caused by kidney stones, tumors, scar tissue, hyperplasia of the prostate, and congenital malformations [14, 16, 17]. Renal fibrosis is the final stage of most progressive kidney diseases. It is induced following inflammation due to autoimmune diseases and inflammatory responses to AKI, and in chronic kidney diseases and diabetic kidney disease. Interstitial fibrosis is often associated with a loss of tubules [5, 18].

Diagnostic tools that enable direct, noninvasive detection of TVF changes are lacking. When TVF changes are induced by changes in GFR, these can be roughly estimated from GFR measurements. GFR can be measured by classical clearance techniques; however, routine clinical diagnostics still rely on the serum concentration of creatinine and/or cystatin C [19]. These surrogate parameters for GFR have notoriously low sensitivity and a rather sluggish response to acute drops in GFR [19]. Obstructions of the extrarenal urinary tract are clinically diagnosed by ultrasonographic or X‐ray urography, yet these methods do not enable assessment of potential concomitant increases in the TVF [13, 20]. Renal core needle biopsies are used to assess fibrosis and edema, among other markers of kidney pathology [21]. However, biopsy specimens usually contain mostly cortical tissue and are limited by inherent drawbacks, including sampling bias and reliance on qualitative, subjective clinical interpretation [21]. Moreover, biopsies are invasive, risking damage to an already compromised kidney and have a nonnegligible rate of complications [22].

Current state‐of‐the‐art techniques for visualizing the tubular lumen with histology or intravital imaging using multiphoton microscopy require invasive procedures, making them entirely unfeasible for longitudinal preclinical studies or eventual clinical use. Intravital microscopy is typically restricted to superficial, localized regions of the kidney [23]. Magnetic Resonance Imaging (MRI) is a nondestructive technique that has proven invaluable for structural and functional nephrology [24, 25, 26, 27]. Although the spatial resolution of MRI is on the mesoscopic scale, ranging from about 20 μm (preclinical) to the millimeter range (clinical), it allows the study of cellular‐level tissue microstructure and tissue water compartments. By utilizing the biophysics behind the MR transversal relaxation time *T*_2_, MRI provides a window into the TVF. *T*_2_ cartography is a quantitative MRI approach used to generate a spatial map of *T*_2_ throughout a field of view. This method has been used to map the myelin water fraction in cerebral white matter in vivo [28, 29]. The water‐containing compartments of renal tissue include (i) the intracellular space, (ii) the interstitial space, (iii) the lumen of the intrarenal vasculature with flowing blood, and (iv) the tubular lumen with flowing tubular fluid—a compartment unique to the kidney. In the kidney, the parenchyma and blood compartments exhibit similar *T*_2_ relaxation times. The tubular fluid has a considerably longer *T*_2_ relaxation time than the parenchyma and blood compartments. The contributions of the slow and fast *T*_2_ relaxation components to the *T*_2_ decay of the MR signal can be decomposed to provide useful information on the renal microstructure; the amplitude of the long *T*_2_ component is a surrogate of the TVF [30, 31].

Our recent work demonstrated proof‐of‐principle of dynamic mapping of the MRI relaxation time *T*_2_ for TVF cartography in rats [31]. This approach facilitated parametric mapping of TVF obtained in vivo under baseline conditions, and upon a clinically realistic acute intervention that increased renal pelvis and tubule pressure [31]. The TVF response to intravenous administration of furosemide has not been explored so far.

Seizing this opportunity, this study focuses on MR cartography to reveal alterations in the renal tubule volume fraction during a diuretic intervention using furosemide. In this work, we define TVF as the internal volume of the tubular lumen that varies with changes in the tubular fluid volume, not the total volume that includes the tubular epithelial cells. We hypothesize that furosemide increases the TVF in rat kidneys. To test this hypothesis, we conducted simultaneous assessment of the TVF derived from the *T*_2_ decay of the MR signal and of kidney size obtained from *T*_2_‐weighted MR images. In parallel, we performed spatially resolved quantification of the effective transversal relaxation time *T*_2_*, which is an MRI surrogate of renal oxygenation. We also examined the total hemoglobin concentration (HbT), which is a surrogate of the renal blood volume fraction, using near‐infrared spectroscopy (NIRS). Changes in TVF, kidney size, and HbT were examined to explore confounding effects on MRI‐based assessment of renal oxygenation.

## 2 Results

### 2.1 Spectral Analysis of the *T*_2_ Relaxation of Renal Parenchyma and Tubular Fluid

**FIGURE 1:**
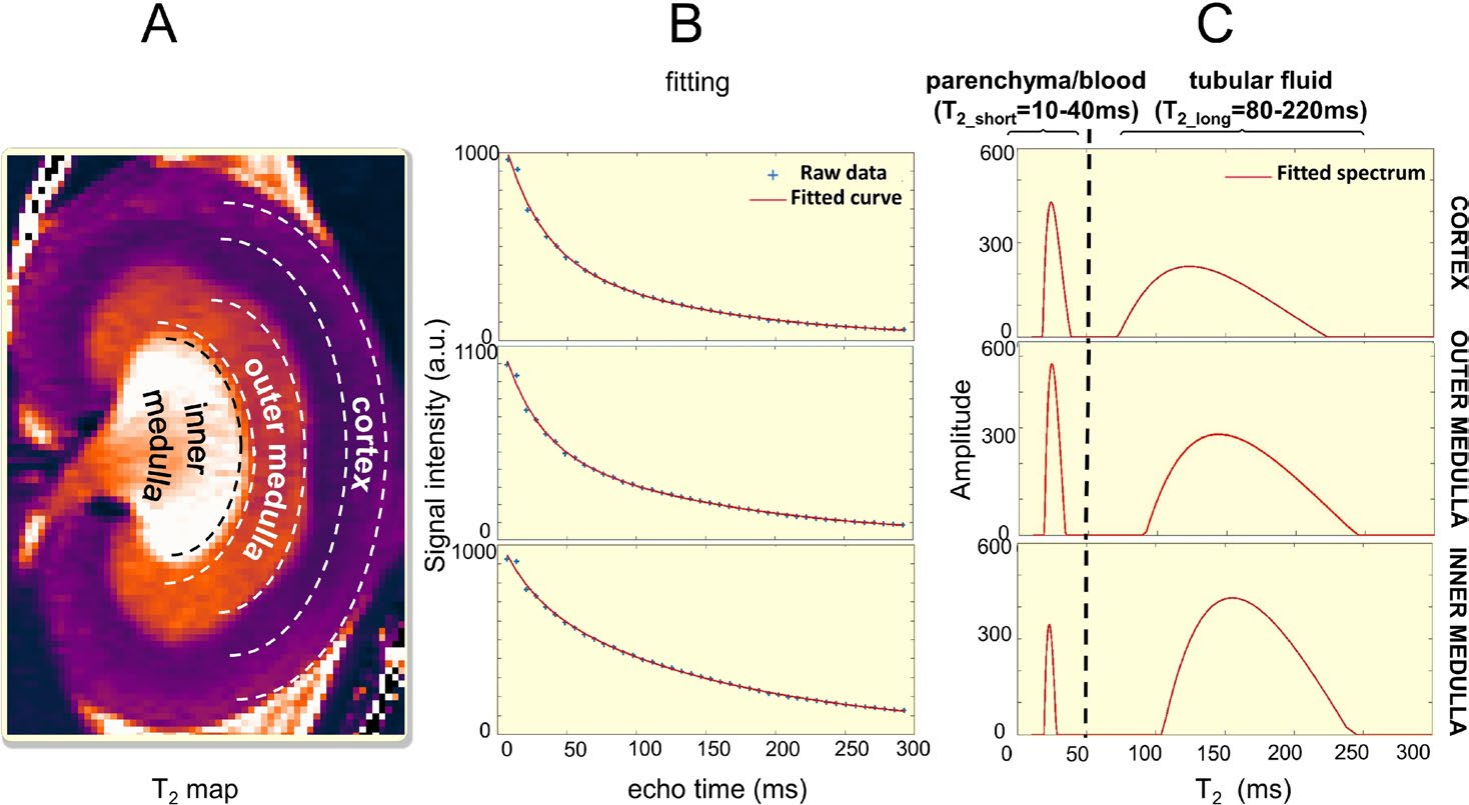
Spectral analysis of the relaxation time *T*_2_ using a free fit with the nonnegative least squares (NNLS) algorithm. (A) In vivo *T*_2_ map of a rat kidney. For spectral analysis of *T*_2_ relaxation regions of interest (ROI) were defined for the renal cortex, the outer medulla and the inner medulla. (B) Representative example of the *T*_2_ fitting for the regions of interest highlighted in A with NNLS. (C) *T*_2_ spectrum obtained for a rat kidney using the ROIs highlighted in A. For data acquisition a minimum echo time of TE = 6.96 ms (ΔTE = 6.96 ms, number of echoes = 42 TR = 2000 ms) was used.

To evaluate the decomposition of *T*_2_ relaxation into short and long *T*_2_ water fractions, we performed data‐driven spectral analysis of the renal *T*_2_ decay using a free fit with nonnegative least squares (NNLS) approach. In vivo data were acquired from two rats (*n* = 2) using a *T*_2_ mapping protocol with an extended echo time range (defined as long TE range protocol, Table 1). This approach was used to determine the number of water compartments, quantify the *T*_2_ relaxation time of rat tubular fluid (*T*_2_long_), and examine the short *T*_2_ relaxation time (*T*_2_short_). Figure 1 shows a representative example of a *T*_2_ spectrum in a rat kidney for the renal cortex (CO), outer medulla (OM) and inner medulla (IM). The analysis of the *T*_2_ decay yielded two peaks, one peak representing the parenchyma and blood compartments, and the other peak representing the tubular fluid. The parenchyma and blood compartments showed a *T*_2_ distribution of *T*_2_short_ = 10–40 ms. The tubular fluid had a *T*_2_ distribution of *T*_2_long_ = 80–220 ms. The detection of the *T*_2__short peak is consistent with previous reports of *T*_2_ ≈40 ms for arterial blood, *T*_2_ ≈41 ms for the renal cortex, and *T*_2_ ≈52 ms for the outer medulla at 9.4 T [32, 33]. Therefore, we ascribed the *T*_2_short_ to parenchyma and renal blood. Prior studies have reported a *T*_2_ value of approximately 180 ms for mouse urine at 9.4 T [34], however, there are no published reports on *T*_2_ relaxation times of rat tubular fluid at 9.4 T. To close this gap and further validate the results obtained from the spectral analysis of the *T*_2_ decay, we measured *T*_2_ = 150 ± 45 ms of rat urine collected from the bladder at body temperature *T* = 37°C (*n* = 2, pH = 6.0). This finding is in accordance with the second peak obtained from the spectral analysis and is within this peak's *T*_2_ distribution of *T*_2_long_ = 80–220 ms, and hence represents the tubular fluid. Assuming that substances typically present in the tubular fluid of healthy subjects do not substantially influence *T*_2_long_ [33], the relaxation time is expected to remain relatively constant across different renal layers. Accordingly, for in vivo TVF assessment, the value of *T*_2_long_ = 150 ms was fixed as the long component of a bi‐exponential nonlinear fit of the *T*_2_‐relaxation driven MR signal decay in the short TE range protocol. *T*_2_short_ was constrained between 10 ms and 40 ms.

**TABLE 1:** Synopsis of the parameters used for the MRI protocols deployed for *T*_2_*, *T*_2_ mapping, and for kidney size estimation.

Method	*T*_2_ mapping		*T*_2_* mapping
	Long‐TE range protocol	Short‐TE range protocol	Multi gradient‐echo
	Multi spin‐echo	Multi spin‐echo	
Repetition time TR (ms)	2000	Prospective triggering minimum was set to 500	50
Number of echoes	42	13	10
First echo time TE_1_ (ms)	6.96	6.4	2.1
Inter‐echo time ΔTE (ms)	6.96	6.4	2.1
Flip angle: *α*_refocusing pulse_(°) or *α*_excitation_(°)	180°	180°	16°
Number of averages NA	1	1	4
Acquisition time *t*_acq_. (s)	170	58	23
In‐plane spatial resolution w/o zero filling (μm^2^)	226 × 445	226 × 445	226 × 445
Field of view (mm^2^)	38.2 × 50.3	38.2 × 50.3	38.2 × 50.3
Matrix size	169 × 215	169 × 215	169 × 215

### 2.2 Validation of TVF Assessment in Phantom Study

**FIGURE 2:**
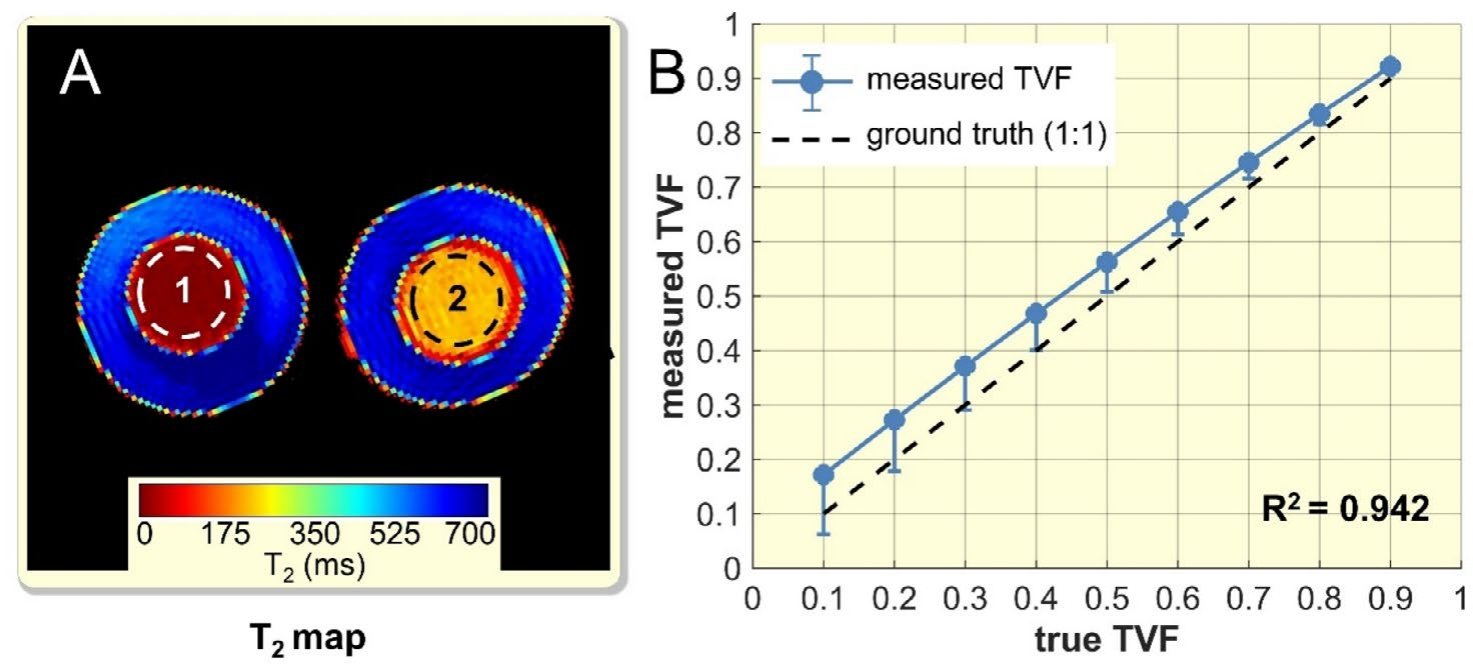
Evaluation of absolute TVF values in a phantom study: (A) *T*_2_ map in milliseconds of the phantom scanned with the short TE protocol using multi‐echo spin‐echo (TR = 500 ms, number of echoes = 13, first TE = 6.4 ms, inter‐echo time ΔTE = 6.4 ms, number of averages = 1, *α*_refocusing pulse_ = 180°, *t*_acquisition_ = 58 s) with selected region‐of‐interest (ROI1–ROI2). ROI1 has a *T*_2_ distribution similar to the *T*_2_ bandwidth of blood/parenchyma. ROI2 has a *T*_2_ distribution similar to the *T*_2_ bandwidth of tubular fluid. The outer tubes are filled with distilled water used as a reference. (B) Evaluation of the assessment of the absolute volume fraction with decomposition of parametric *T*_2_ using bi exponential fitting of the *T*_2_ decay with fixed *T*_2_long_. The coefficient of determination *R* ^2^ of 0.942 indicates a strong agreement for TVF assessments.

Validation of TVF assessment including absolute TVF values was done using a phantom containing rat urine with known volume fractions. Figure 2A shows a *T*_2_ map obtained for the phantom. The inner tube of the left phantom (ROI1) has a *T*_2_ distribution of 58–69 ms, mimicking the *T*_2_ relaxation times of the renal blood/parenchyma. The inner tube of the right phantom (ROI2) has a *T*_2_ distribution of 180–260 ms, mimicking the *T*_2_ relaxation time of tubular fluid. A single MRI voxel in the kidney can contain multiple, isolated water compartments. We simulated this by assuming the summation of the signals measured in the ROI1 and ROI2 in Figure 2A arises from the same voxel. The two ROIs contain nearly identical volumes of water, so the proton populations should be nearly identical, and the true relative fractions (ground truth) should be ≈50%. Similarly, by scaling the signal measured from the left and right ROI, we can simulate the tubule water fraction for different ranges. Figure 2B shows absolute TVF estimated with a bi‐exponential fit (*T*_2_short_ lower band = 50 ms—*T*_2_short_ upper band = 80 ms. *T*_2_long_ fixed = 220 ms). For the TVF assessment, a mean absolute error (MAE) of 5.6% ± 1.8% and an adjusted *R* ^2^ of 0.942 were observed.

### 2.3 Validation of TVF Assessment With Synthetic Data

We simulated *T*_2_ decays to examine the accuracy of our bi‐exponential fit with a fixed *T*_2_long_ on the TVF assessment. Table 2 summarizes the impact of fixing *T*_2_long_ at 150 ms on the assessment of absolute TVF and relative changes in TVF. Relative changes refer to the difference in TVF between two consecutive points (n, n‐1), expressed as a percentage of the value of the previous point. Since the relative changes remain fairly similar to ground truth across a wide range of TVFs, the error in the relative changes is sufficiently small despite the inherent bias in the absolute TVF values (Table 2).

**TABLE 2:** Representative mean absolute error (MAE) for TVF relative changes, which were derived from analysis of the simulated *T*_2_ decays using bi‐exponential fitting, covering a broad physiological range of TVFs. During the fitting process, (left column) *T*_2___long_ was incorrectly fixed at 150 ms, which is shorter than its actual simulated value of 200 ms. (center column) *T*_2___long_ was correctly fixed at 150 ms, which is equal to its actual simulated value of 150 ms. (right column) *T*_2___long_ was incorrectly fixed at 150 ms, which is longer than its actual simulated value of 100 ms. Other parameters of the simulation are identical with those used for TVF assessment in vivo MRI study first TE = 6.4 ms, inter‐echo time ΔTE = 6.4 ms, number of echoes = 13, *α*_refocusing_pulse_ = 170° (to account for imperfect refocusing pulses).

*n*	Ground truth		*T*_2_long_ incorrect fixation (underestimated)			*T*_2_long_ correct fixation			*T*_2_long_ incorrect fixation (overestimated)		
			Ground truth simulated with *T*_2_long_ = 200 ms			Ground truth simulated with *T*_2_long_ = 150 ms			Ground truth simulated with *T*_2_long_ = 100 ms		
			*T*_2_long_ set to 150 ms during fitting			*T*_2_long_ set to 150 ms during fitting			*T*_2_long_ set to 150 ms during fitting		
	TVF (%)	TVF relative change (%)	TVF (%)	TVF relative change (%)	MAE for relative change (%)	TVF (%)	TVF relative change (%)	MAE for relative change (%)	TVF (%)	TVF relative change (%)	MAE for relative change (%)
1	**20.0**	**33.3**	23.9 ± 0.6	34.3	2.0	21.6 ± 1.3	30.1	5.0	12.8 ± 0.8	34.7	4.7
2	**25.0**	**25.0**	29.9 ± 0.6	25.1	1.6	26.6 ± 1.3	23.1	4.2	16.0 ± 0.8	25.0	4.2
3	**30.0**	**20.0**	35.9 ± 0.5	20.1	1.3	31.5 ± 1.3	18.4	3.6	19.2 ± 0.9	20.0	3.9
4	**35.0**	**16.7**	41.9 ± 0.5	16.7	1.1	36.5 ± 1.3	15.9	3.1	22.4 ± 0.9	16.7	3.6
5	**40.0**	**14.3**	47.8 ± 0.5	14.1	0.9	41.5 ± 1.3	13.7	2.8	25.5 ± 1.0	13.8	3.5
6	**45.0**	**12.5**	53.6 ± 0.5	12.1	0.8	46.4 ± 1.3	11.8	2.5	28.5 ± 1.0	11.8	3.5
7	**50.0**	**11.1**	59.4 ± 0.5	10.8	0.8	51.4 ± 1.2	10.8	2.3	31.5 ± 1.1	10.5	3.6
8	**55.0**	**10.0**	65.2 ± 0.5	9.8	0.9	56.4 ± 1.2	9.7	2.1	34.3 ± 1.3	8.9	3.8
9	**60.0**	**9.1**	70.8 ± 0.5	8.6	0.9	61.3 ± 1.2	8.7	1.9	37.0 ± 1.4	7.9	4.2
10	**65.0**	**8.3**	76.4 ± 0.6	7.9	0.8	66.3 ± 1.1	8.2	1.7	39.5 ± 1.6	6.8	4.7
11	**70.0**	**7.7**	82.1 ± 0.5	7.5	0.7	71.2 ± 1.1	7.4	1.5	41.8 ± 1.8	5.8	4.9
12	**75.0**	**7.1**	87.7 ± 0.5	6.8	0.6	76.1 ± 1.1	6.9	1.4	44.0 ± 1.7	5.3	3.8
13	**80.0**	**6.7**	93.5 ± 0.5	6.6	1.5	81.0 ± 1.0	6.4	1.3	46.9 ± 1.3	6.6	3.2

*Note:* The bold values in Table 2 are ground truth and predefined simulation parameters and therefore contain no error.

### 2.4 Tubular Volume Fraction Changes Upon Furosemide Application

**FIGURE 3:**
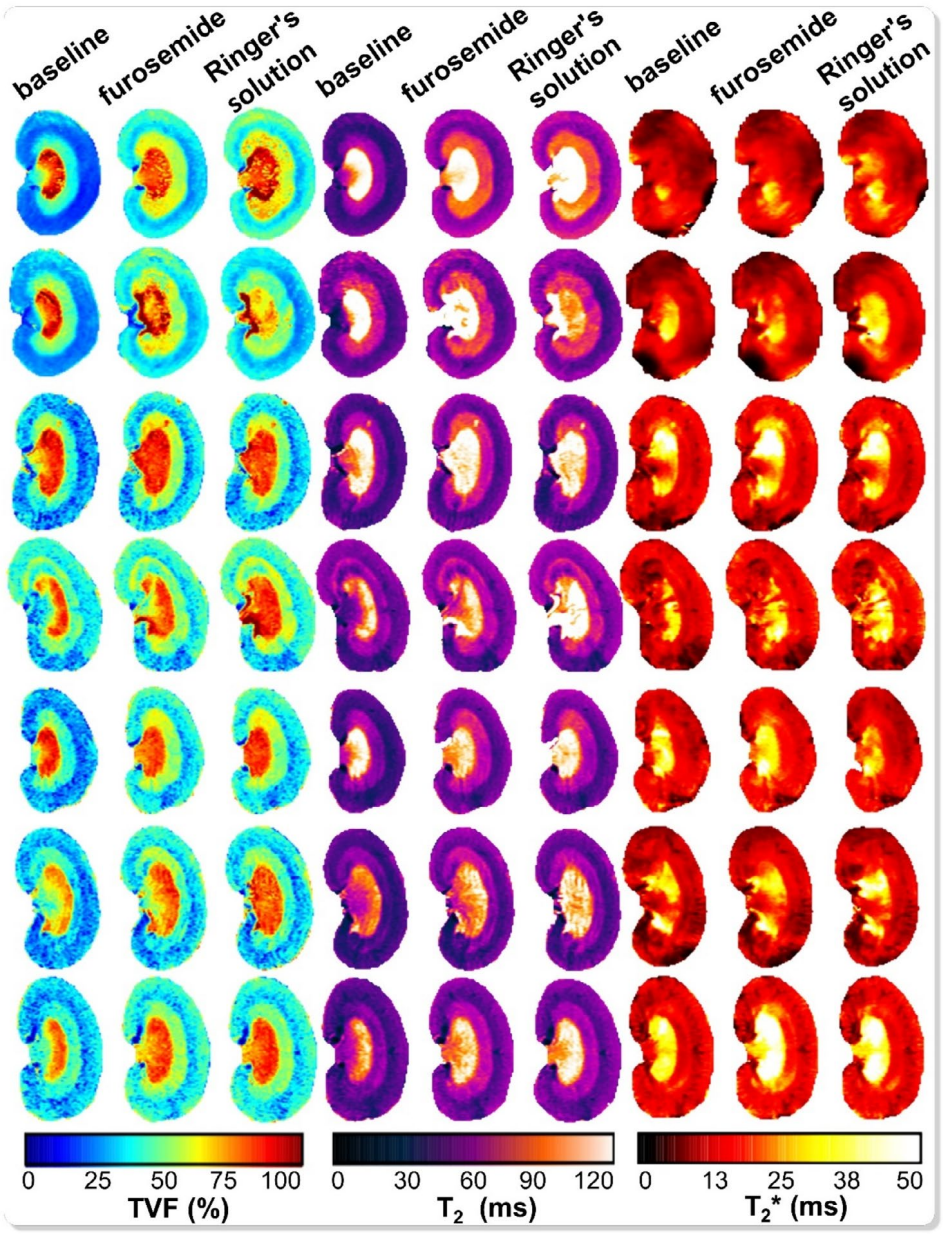
Cartography of the tubular volume fraction TVF and the MR relaxation times *T*_2_ and *T*_2_* obtained in rat kidneys in vivo at baseline, ~4 min after furosemide injection, and ~18 min after furosemide injection ~6 min after the start of the infusion of the electrolyte solution (Ringer's solution). Marked alterations in TVF and *T*_2_ following furosemide injection are visually evident. Postinjection, an increase in TVF was observed in both the renal cortex and outer medulla. Similarly, *T*_2_ values exhibited a notable rise in the renal cortex and outer medulla. Additionally, a marked increase in *T*_2_* was observed in the outer medulla.

**FIGURE 4:**
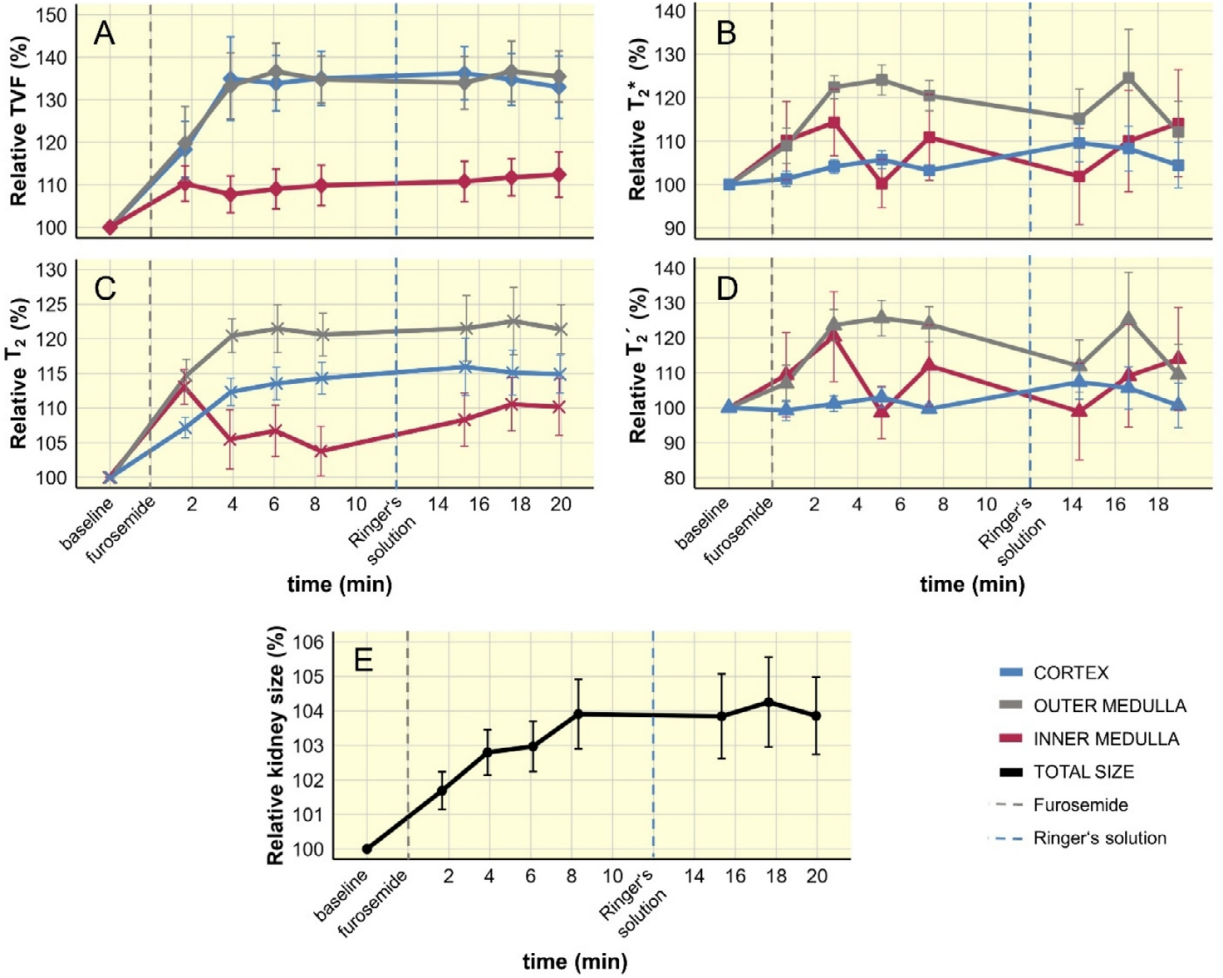
Time courses of relative changes following administration of furosemide. (A) Time course of TVF changes (mean ± SEM, *n* = 7) for cortex (blue), outer medulla (gray), inner medulla (red) before the intervention (baseline), after the furosemide administration (gray dashed line) and during the infusion of Ringer's solution (blue dashed line), (B) time course of *T*_2_* changes for cortex, outer medulla, inner medulla, (C) time course of *T*_2_ changes for cortex, outer medulla, inner medulla, (D) time course of *T*_2_´ changes for cortex, outer medulla, inner medulla, and (E) time course of kidney size changes before (baseline) and during these interventions. Gray dashed line at time = 0 indicates the start of the furosemide injection. Blue dashed line at time = 12 min indicates the start of the infusion of the electrolyte solution (Ringer's solution). Time points at *t* = 2 min, 4 min and 6 min are reported as mean ± SEM of *n* = 6 rats. This is due to the different time point of acquisition in the first two rats, which underwent MR scans using both the long TE range and short TE range protocols.

Representative TVF maps were derived from bi‐exponential decomposition of *T*_2_ decays obtained from rats in vivo (Figure 3, left). Data was acquired during baseline, and during the first interval (1–10 min) after intravenous bolus injection of furosemide followed by a saline chaser (to ensure that the entire furosemide dose entered the circulation). Data was then acquired during a second interval (12–20 min), when a balanced electrolyte solution (Ringer's solution) was continuously infused to replace the volume and electrolyte loss caused by furosemide. Under baseline conditions, TVF values were TVF_CORTEX_ = 29.8% ± 2.2% (mean ± SEM, *n* = 7), TVF_OUTER_MEDULLA_ = 42.6% ± 2.9%, and TVF_INNER_MEDULLA_ = 76.4% ± 3.7%. Figure 4A shows the time courses of relative changes in TVF in response to furosemide and the subsequent infusion of Ringer's solution. TVF changes were immediately observed after furosemide injection. Averaged over the four time points of the first time interval, TVF_CORTEX_ increased by 31.1% ± 6.3% compared to baseline. Averaged over the three time points of the second time interval, TVF_CORTEX_ remained increased by 33.8% ± 5.7% vs. baseline. A similar effect was observed in the OM, where TVF increased by 30.7% ± 6.2% during the first interval and remained increased by 34.8% ± 5.6% during the second interval. In the IM, our data suggest a slight increase of TVF by 8.8% ± 3.8% in the first interval and by 11.7% ± 4.4% in the second interval, although these results were not statistically significant.

**FIGURE 5:**
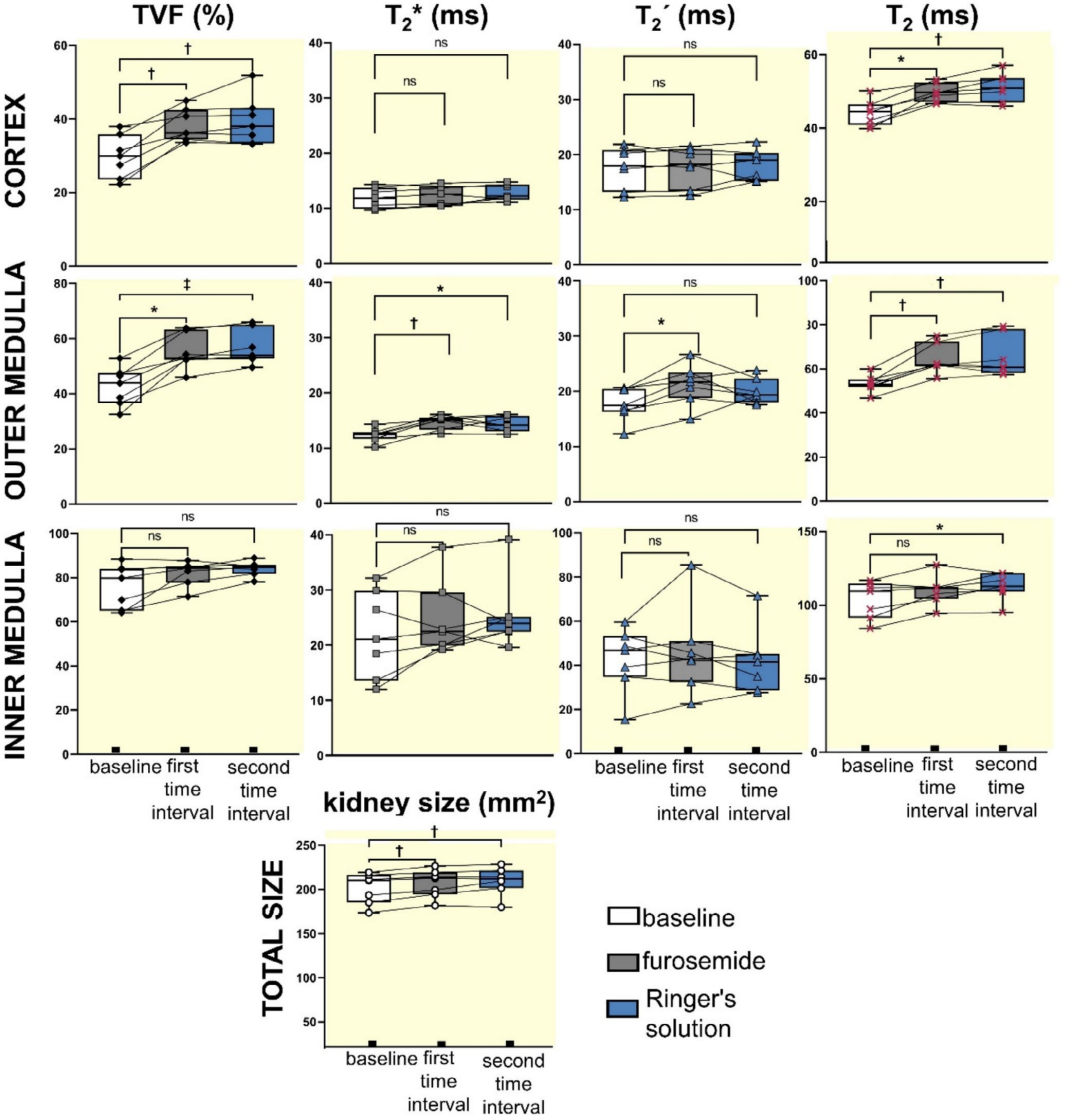
Changes in TVF, *T*_2_*, *T*_2_´, *T*_2_ and kidney size following administration of furosemide. The TVF showed significant changes in response to furosemide in the renal cortex and outer medulla, but not in the inner medulla. Pair‐wise comparisons show significant increases compared to baseline during the furosemide intervention and the Ringer's solution administration in the cortex (*p* = 0.0075, *p* = 0.0033, respectively) and outer medulla (*p* = 0.0325, *p* = 0.0005). *T*_2_* was significantly increased in the outer medulla during the furosemide intervention and Ringer's solution administration (*p* = 0.0075, *p* = 0.0325). *T*_2_* changes were not significantly changed in the renal cortex and inner medulla. *T*_2_´ was significantly increased only in the outer medulla during the furosemide intervention (*p* = 0.0272). *T*_2_ was significantly increased during the furosemide intervention and Ringer's solution administration in the cortex (*p* = 0.0162, *p* = 0.0013) and outer medulla (*p* = 0.0075, *p* = 0.0033). In the inner medulla, *T*_2_ was significantly increased only during the Ringer's solution administration (*p* = 0.0162). Kidney size significantly increased during the intervention and upon Ringer's solution administration (*p* = 0.0075, *p* = 0.0033). Nonparametric repeated‐measures Friedman test, with Dunn's post hoc test for pair‐wise comparisons; *n* = 7;*p < 0.05, †*p* < 0.01, ‡*p* < 0.001.

**FIGURE 6:**
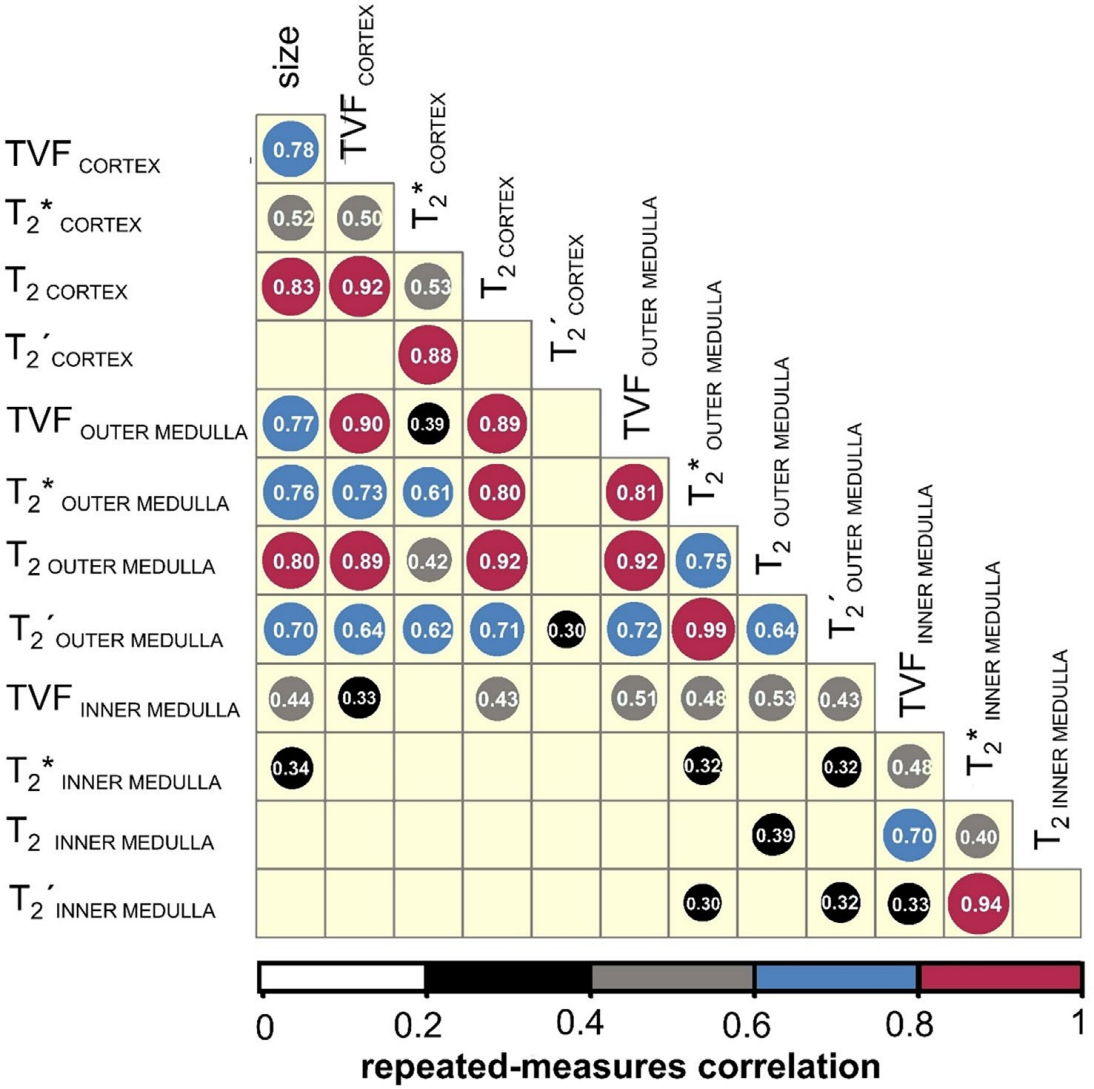
Repeated‐measures correlation matrix illustrating the association between changes in TVF, kidney size, *T*_2_*, *T*_2_´, *T*_2_ for the renal cortex, the outer medulla and the inner medulla. The color legend and the circle size indicate the strength of the correlation. Numbers inside circles indicate correlation coefficients; *n* = 7. Nonsignificant correlations are omitted.

Statistical analyses by ANOVA documented significant changes in TVF. Pair‐wise comparisons showed that TVF_CORTEX_ was significantly increased versus baseline during the first interval (*p* = 0.0075, Figure 5) and remained significantly higher than baseline during the second interval (*p* = 0.0033). Pair‐wise comparison of TVF_OUTER_MEDULLA_ showed a significant increase between baseline and the first interval (*p* = 0.0325, Figure 6) and the second interval (*p* = 0.0005). The pair‐wise comparisons of TVF_INNER_MEDULLA_ did not show significant changes between baseline and the first interval (*p* = 0.5443) or the second interval (*p* = 0.1841).

### 2.5 *T*_2_ * Changes Upon Furosemide Application

For MRI‐based assessment of renal oxygenation, quantitative in vivo *T*_2_* maps were generated during baseline, after furosemide application followed by the saline chaser (1–10 min), and the second interval (12–20 min) during the infusion of the balanced electrolyte solution (Figure 3, right). Under baseline conditions, *T*_2_*_CORTEX_ was 11.9 ± 0.7 ms, *T*_2_*_OUTER_MEDULLA_ 12.3 ± 0.5 ms, and *T*_2_*_INNER_MEDULLA_ 22.0 ± 3.0 ms (*n* = 7). The *T*_2_* time course is shown in Figure 4B. Furosemide led to a significant *T*_2_* increase in the OM by 20.5% ± 3.9% (*p* = 0.0075) during the first interval (averaged over 4 time points) and remained increased by 19.2% ± 7.4% (*p* = 0.0325) in the second interval (averaged over 3 time points), but not in CO or IM (Figure 5). Changes in *T*_2_*_OUTER_MEDULLA_ correlated strongly with changes in TVF_OUTER_MEDULLA_ (*r* = 0.81), while *T*_2_*_CORTEX_ showed moderate correlations with TVF_CORTEX_ (*r* = 0.5; Figure 6).

### 2.6 *T*_2_ Changes Upon Furosemide Application

Quantitative *T*_2_ relaxation maps were derived from mono‐exponential fits (Figure 3, center). Under baseline conditions, *T*_2_ was 44.1 ± 1.3 ms for CO, 53.3 ± 1.5 ms for OM, and 103.6 ± 4.8 ms for IM (*n* = 7). The *T*_2_ time course is presented in Figure 4C. After furosemide injection, *T*_2CORTEX_ increased by 13.0% ± 2.3% during the first interval (averaged over 4 time points) and remained increased by 16.1% ± 3.6% in the second interval (averaged over 3 time points). *T*_2OUTER_MEDULLA_ increased by 20.6% ± 2.7% in the first interval and remained increased by 22.7% ± 4.5% in the second time interval. *T*_2INNER_MEDULLA_ responses were inconsistent. Statistical analysis showed significant *T_2_* changes in response to furosemide for CO (*p* = 0.0012) and OM (*p* = 0.0027), but not for IM (*p* = 0.0515) (Figure 5). *T*_2_ changes in CO and OM strongly correlated with changes in TVF_CORTEX_ and TVF_OUTER_MEDULLA_ (*r* = 0.92); *T*_2CORTEX_ showed moderate correlation with *T*_2_*_CORTEX_ (*r* = 0.53). *T*_2OUTER_MEDULLA_ showed strong correlation with *T*_2_*_OUTER_MEDULLA_ (*r* = 0.75; Figure 6).

### 2.7 *T*_2_ ´ Changes Upon Furosemide Application

Under baseline conditions, *T*_2_ **´** was 17.7 ± 1.4 ms for CO, 17.8 ± 1.3 ms for OM, and 18.3 ± 1.0 ms for IM (*n* = 7). The *T*_2_ **´** time course is presented in Figure 4D. Furosemide only led to a significant *T*_2_ **´** increase in the OM by 20.2% ± 3.6% (*p* = 0.0272) during the first interval (averaged over 4 time points) (Figure 5). Changes in *T*_2_ **´** correlated strongly with changes in *T*_2_* in CO, OM, and IM (*r* = 0.88, 0.99, and 0.94, respectively; Figure 6).

### 2.8 Kidney Size Changes Upon Furosemide Application

Kidney size (KS) showed significant changes in response to the furosemide intervention (Figure 4F). During baseline, the KS was 201 ± 17.4 mm^2^ (mean ± SEM, *n* = 7). After furosemide injection, KS increased by 2.9% ± 0.6% during the first interval (averaged over 4 time points) and remained increased by 4.2% ± 1.2% in the second interval (averaged over 3 time points). Pair‐wise comparisons revealed a significant increase in KS between baseline and the first time interval (*p* = 0.0075) and remained significantly increased during the second interval (*p* = 0.0033) (Figure 5). KS changes correlated strongly with changes in *T*_2 CORTEX_ and *T*_2 OUTER MEDULLA_ (*r* = 0.83 and 0.80, respectively). KS changes correlated strongly with changes in TVF_CORTEX_ and TVF_OUTER MEDULLA_ (*r* = 0.78 and 0.77, respectively). KS changes showed moderate correlation with *T*_2_*_CORTEX_ (*r* = 0.52) and a strong correlation with *T*_2_*_OUTER MEDULLA_ (*r* = 0.76; Figure 6).

### 2.9 Total Hemoglobin Concentration Changes Upon Furosemide Application

**FIGURE 7:**
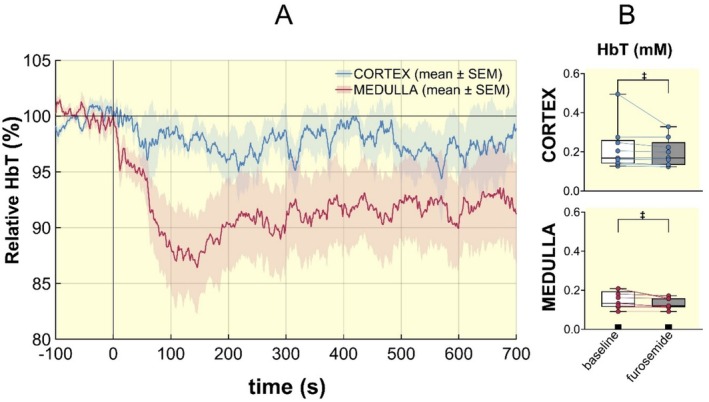
Changes in total hemoglobin concentration (HbT), a surrogate of renal blood volume fraction, obtained from near infrared spectroscopy (NIRS) in the renal cortex (blue) and medulla (red) following furosemide injection (*n* = 9). (A) Time course of relative HbT values are shown as percentage change relative to baseline. The vertical line indicates the time point of furosemide administration (*t* = 0 s). Shaded areas represent the standard error of the mean (SEM). (B) Furosemide administration led to a significant decrease from baseline in the cortex and medulla (*p* < 0.0001), assessed using a paired nonparametric Friedman test.

Near Infrared Spectroscopy (NIRS) was performed to assess changes in the total hemoglobin concentration, a surrogate of renal blood volume, upon furosemide application. Representative time courses showing relative changes in cortical and medullary total hemoglobin concentration (HbT) in response to intravenous furosemide administration in rats (*n* = 9) are presented in Figure 7. In the cortex, furosemide induced a modest but consistent decrease in HbT, with an average reduction of 2.7% ± 0.1% postinjection (*p* < 0.0001). In the medulla, HbT declined more prominently, with an average reduction of 8.6% ± 0.1% following furosemide administration. This was followed by a sustained plateau phase and remained significantly below baseline throughout the entire observation period of 12 min. Pairwise comparisons revealed a significant decrease in HbT between baseline and post furosemide administration (*p* < 0.0001). The reduction in HbT is indicative of a reduction in the renal blood volume fraction related to the concomitant TVF increase.

## 3 Discussion

In this study, we applied bi‐exponential analysis of the MRI *T*_2_‐decay to report for the first time in vivo changes of the tubular fluid volume fraction within the rat kidney in response to furosemide. Our approach adds an innovative physiometabolic dimension to imaging and provides a technical foundation and springboard for research into physiological and pathophysiological conditions of the renal tubular system. Due to the noninvasive nature, MR cartography of the TVF facilitates longitudinal studies and swift translation from preclinical research to human studies and clinical application.

We employed a novel approach and applied spectral analysis of the renal *T*_2_ relaxation decay in vivo. This analysis was conducted to determine the number of water compartments in the kidney and to assess the *T*_2_ relaxation times of rat tubular fluid and of the short *T*_2_ component. Detection of the *T*_2_short_ peak derived from the spectral analysis aligns with previous 9.4 T findings using mono‐exponential fitting: *T*_2 ARTERIAL BLOOD_≈40 ms, *T*_2 CORTEX_≈41 ms, and *T*_2 OUTER MEDULLA_≈52 ms [32, 35, 36]. We therefore attributed *T*_2_short_ to the renal parenchyma and blood. While mouse urine *T*_2_ at 9.4 T has been reported [34], data for rat tubular fluid were lacking. To address this, we measured *T*_2_ in rat urine, which is consistent with the *T*_2_long_ peak derived from the spectral analysis, supporting its assignment to tubular fluid. Validation of the absolute TVF values was done using a phantom containing rat urine and material mimicking the relaxation properties of renal tissue with known volume fractions. The coefficient of determination *R* ^2^ of 0.942 indicates a strong agreement for TVF assessments. Additionally, we conducted simulations to explore potential errors in TVF assessment that could arise from using short TE range protocols and fixation of the *T*_2_long_ value. The simulation results indicated that if *T*_2_long_ is fixed to an incorrect value, either below or above the true value, the absolute TVF could be misestimated. However, the relative changes in TVF remained consistent and predictable.

To investigate the effects of furosemide induced TVF changes in the rat kidney, we administered an intravenous bolus directly while the rats were in the MRI scanner. Furosemide inhibits the NKCC2 transporter in the thick ascending limb of the loop of Henle, which results in decreased water reabsorption in the nephron portions distal to the thick ascending limb. Our findings on TVF dynamics reflect this effect. We noted a significant TVF increase in the renal cortex (TVF_CORTEX_) and the outer medulla (TVF_OUTER_MEDULLA_). A slight increase in the inner medulla was not statistically significant (TVF_INNER_MEDULLA_). We demonstrated this method can be used to quantify acute dynamic changes in TVF. Therefore, MR‐TVF cartography can be a valuable tool to investigate the mechanisms and assess the severity of certain renal diseases, particularly in the context of acute changes in TVF.

By inhibiting energy‐dependent electrolyte reabsorption, furosemide reduces oxygen consumption in the thick ascending limb of the loop of Henle, which in turn elevates tissue oxygen levels, particularly in the OM. This was reflected by our observation of increases in the MRI relaxation times *T*_2_* and *T*_2_, which are both surrogates of blood oxygenation [27]. While our current observations of *T*_2_* and *T*_2_ align with prior studies, the observed increases in *T*_2_*, *T*_2_ are not solely due to improved oxygenation, as has hitherto generally been supposed [37, 38, 39, 40, 41, 42, 43, 44, 45]. The markedly more pronounced increase in TVF compared to the increase in kidney size is indicative of an increase in intrarenal pressure. Substantial increases in intratubular pressure following the administration of furosemide as well as another diuretic, acetazolamide, in rats have previously been reported [46, 47, 48]. Because the renal capsule is relatively rigid, intrarenal pressure will increase. This results in intrarenal compartment syndrome: intrarenal blood vessels become compressed and the renal blood volume fraction (BVF) is therefore reduced when the intrarenal pressure increases [14, 15]. Our findings derived from NIRS demonstrate furosemide‐induced reduction in the BVF: the concentration of total hemoglobin per tissue volume, a surrogate parameter for BVF, was markedly decreased, especially in the renal medulla.

We observed an increase in TVF in response to furosemide administration. This change was similar for the renal cortex and outer medulla. The *T*_2_* increase in the cortex was much smaller than in the outer medulla. This observation is consistent with results obtained in healthy subjects: furosemide injection resulted in about a 50% increase in medullary *T*_2_*, while cortical *T*_2_* was barely changed [37, 49]. Four effects contribute to this phenomenon. First, a considerable portion of the cortical vessels are arteries, arterioles, and veins, and their vessel walls will withstand the compression better than the comparatively supple walls of the microvessels in the medulla. Second, the average oxygen saturation of the hemoglobin in the cortical vessels is typically higher than in the medulla [50], leading to a weaker deoxyhemoglobin reduction even if the blood volume fraction reduction was similar. Third, the absolute value of the tubular volume fraction is smaller in the cortex. This means a similar relative change relates to a smaller absolute change. Fourth, changes in the total hemoglobin concentration (HbT), a surrogate of the BVF, were much more pronounced in the medulla than in the renal cortex, as demonstrated by our NIRS measurements.

The MRI relaxation times *T*_2_ and *T*_2_* are sensitive to the concentration of deoxyhemoglobin (deoxyHb) per tissue volume, so any reduction in BVF increases *T*_2_ and *T*_2_* [27]. Therefore, *T*_2_*, *T*_2_ based assessments of furosemide‐induced changes in renal oxygenation overestimate the gain in true tissue oxygenation, that is, in the tissue partial pressure of oxygen (pO_2_). An early study in rats that utilized invasive probes—the “gold standard” for measuring true tissue oxygenation—reported an increase in medullary pO_2_ upon a large bolus dose of furosemide, while cortical pO_2_ remained unchanged [50]. In order to determine the contribution of the true oxygenation gain versus the effects of the compartment syndrome‐induced changes, concurrent *T*_2_ and *T*_2_*‐based evaluations of tissue oxygenation coupled with measurements from invasive probes are necessary.

It has long been recognized that changes in kidney size are indicative of certain pathophysiologic developments. An increasing body of literature outlines the potential of noninvasive imaging for evaluating kidney size as a clinical parameter in the diagnosis, treatment monitoring, and prognosis in renal disease [51]. A notable example is polycystic kidney disease (PKD), wherein kidney size correlates with disease progression [12, 52, 53]. Consequently, the U.S. Food and Drug Administration and the European Medicines Agency now include kidney size as a prognostic marker for use in clinical trials of new therapies for autosomal dominant PKD [54, 55]. Detecting reduction in kidney size due to parenchymal atrophy, sclerosis, and fibrosis has also been recognized as a tool to identify chronic kidney disease and to determine its severity [56, 57]. Imaging‐based kidney size is currently included as a prognostic imaging marker for diabetic kidney disease [58], and was recently proposed for longitudinal monitoring for several renal diseases including hyperfiltration in early diabetic nephropathy, renal transplants, renal artery stenosis, and vesicoureteral reflux [59]. Preclinical MRI studies emulating various clinical conditions and acute scenarios have documented substantial changes in kidney size. This included clinically relevant scenarios with primary changes in TVF, in BVF, or in both fractions [31, 60, 61, 62, 63, 64, 65, 66, 67, 68, 69, 70, 71]. The results of the current study corroborate that a furosemide‐induced TVF increase is sufficient to result in a small increase in kidney size.

Renal tissue hypoxia is a pivotal early element in the pathophysiology of acute kidney injury and its subsequent progression to chronic kidney disease. Hypoxia also plays a major role in the pathophysiology of diabetic kidney disease [70, 72, 73, 74, 75, 76, 77]. Therefore, assessment of renal oxygenation by *T*_2_* and *T*_2_ MRI could become a vital assay for research into renal (patho‐)physiology and for clinical application. However, as these metrics reflect the amount of deoxyHb per tissue volume, *T*_2_* and *T*_2_ are also dependent on the renal BVF. One cause for changes in renal deoxyHb independent of changes in blood oxygenation is alterations in TVF, because changes in TVF likely induce alterations in renal BVF. Indeed, TVF is recognized as a major confounding factor influencing the relationship between renal *T*_2_* and *T*_2_ and tissue pO_2_ [27, 62, 70, 78, 79, 80, 81]. Recognizing that events leading to acute renal hypoxia are often associated with changes in BVF and/or TVF, and that these changes are accompanied by changes in kidney size, we recently used dynamic MRI to monitor kidney size in parallel with *T*_2_*, *T*_2_ mapping in rats during clinically realistic interventions that alter renal tissue oxygenation. That study demonstrated that monitoring kidney size greatly facilitates the appropriate physiological interpretation of acute renal oxygenation changes obtained by *T*_2_*, *T*_2_ [60]. However, measurements of acute changes in kidney size alone cannot differentiate between changes in the BVF and changes in the TVF. If the reasons underlying a change in kidney size (i.e. changes in TVF or BVF) are not obvious from the respective preclinical or clinical scenario, advanced MR methods that support monitoring of acute changes in BVF and TVF can enable the quantification of their individual contributions to changes in kidney size. MRI‐based measurement of renal BVF currently requires off‐label administration of intravascular contrast agents, which limits broad use of this approach in patients who may not tolerate such agents [60]. Hence, *T*_2_ assessments of changes in the TVF are highly relevant for elucidating the mechanisms of renal pathophysiology and will help to accurately determine the pathophysiological role of changes in renal oxygenation assessed by renal *T*_2_*, *T*_2_ mapping [80].

The vast majority of reports on *T*_2_ mapping in kidney tissue and its association with kidney injury use a mono‐exponential fitting of the *T*_2_ decay [60, 82, 83, 84, 85]. Hence, these studies fail to distinguish between the tubular fluid and blood/parenchyma compartments, and incorrectly attribute changes in *T*_2_. It is standard practice to assume that an MRI voxel consists of homogeneous tissue. However, this is an oversimplification and is especially problematic when imaging renal tissue. A single MRI voxel (the volume element of an image) typically has a size on the order of 0.25 × 0.25 × 1 mm [3] in preclinical studies. Such a voxel contains at least two, and often all four fluid compartments within renal tissue, including the intracellular space, the interstitial space, the lumen of the intrarenal vasculature with flowing blood, and the tubular lumen with flowing tubular fluid. This results in a multi‐exponential *T*_2_ signal decay curve. Therefore, future studies should capitalize on the fact that the *T*_2_ time of water is determined by its varied local environment and use a longer TE range and multi‐exponential analysis to gain a more nuanced understanding of *T*_2_ changes associated with renal tissue.

Our results demonstrate that concomitantly measured MRI data on renal TVF, kidney size, and oxygenation‐dependent MRI metrics enable assessment of rapid changes in the tubular fluid content, the renal blood content, and kidney size in response to physiological and pharmacological stimuli. Our findings highlight the potential for misinterpretation of data from blood oxygenation level‐dependent MRI using *T*_2_ and *T*_2_* mapping, and the significant sources of error when using these MRI metrics to estimate tissue oxygenation in the kidney. The increase in TVF upon furosemide administration and the consequent reduction in renal BVF, resulting from compartment syndrome within the rigid capsule of the kidney, mimics an apparent rise in tissue oxygenation as derived from *T*_2_ and *T*_2_*. Since these metrics mirror the concentration of deoxyHb per tissue volume, and because changes in TVF often induce alterations in renal BVF, altered TVF is a prominent cause of changes in *T*_2_, *T*_2_* that are independent of changes in renal blood oxygenation [62, 80]. Hence, the development, evaluation, and application of methods for TVF measurement are imperative for a correct physiological interpretation of MRI of renal oxygenation.

## 4 Conclusion

Our study demonstrates the potential of TVF cartography to gain deeper insights into short‐ and long‐term effects of drugs or physiological interventions on the renal TVF and renal oxygenation. MR‐based TVF cartography offers a novel approach to investigate the mechanisms of renal disease, and to monitor disease severity and responses to therapy that holds potential promise to ultimately become a clinically impactful noninvasive diagnostic tool.

## 5 Materials & Methods

All submitted materials and data adhere to the good publishing practices outlined in the Acta Physiologica guidelines for physiology research [86].

### 5.1 In Vivo Study Preparations

All experiments were approved by the Animal Welfare Department of the State Office of Health and Social Affairs of Berlin, in accordance with German Animal Protection Law and approved guidelines (permission reference is G0043/19). Male Wistar rats (*n* = 9, aged 12–13 weeks, body mass 270–300 g, Harlan‐Winkelmann, Borchen, Germany) were used. The animals had *ad libitum* access to standard diet and water and were housed under standard conditions with environmental enrichment [60, 63].

Rats underwent surgical procedures involving insertion of vascular catheters and invasive probes for quantitative measurements of renal haemodynamics and oxygenation, as previously described [63]. For anesthesia, urethane (0.2 g/mL in distilled water; 6 mL/kg BM intraperitoneal; Sigma‐Aldrich, Steinheim, Germany) was used throughout the surgical preparation and examination [63, 87, 88, 89, 90]. Urethane provides long‐lasting anesthesia with minimal effects on cardiovascular and respiratory control compared to other anesthetics.

### 5.2 MRI Protocols

MRI was performed on a 9.4 Tesla small animal MR system (Bruker Biospec 94/20, Bruker Biospin, Ettlingen, Germany) using a linear radiofrequency (RF) volume resonator for transmission. For signal reception, a 4‐channel surface RF coil array (Bruker Biospin, Ettlingen, Germany) tailored for rats was deployed [63]. For geometric planning and slice positioning, *T*_2_‐weighted pilot scans were acquired. Local volume selective shimming of the magnetic field homogeneity on a voxel accommodating the left kidney was conducted using an automatic optimization algorithm based on free induction decay length. For *T*_2_* mapping, a multi gradient‐echo (MGE) technique (Table 1) was used. For *T*_2_ mapping, two multi spin‐echo protocols were employed: (i) Long TE range protocol to enable spectral analysis of the renal *T*_2_ decay acquired in vivo from two rats (*n* = 2) at baseline prior to interventions; (ii) Short TE range protocol used for in vivo assessment of *T*_2_ and TVF in rats (*n* = 7) during baseline and intervention [31]. For motion compensation, *T*_2_ and *T*_2_* mapping were performed using respiratory triggering [63]. Details of the MRI parameters for both *T*_2_ mapping protocols and the *T*_2_* protocol are provided in Table 1.

### 5.3 Diuretic Intervention

The interventional study used the following workflow: a ~10 min baseline period with MR data acquisition, followed by an intravenous bolus of furosemide (5 mg/kg, Ratiopharm, Ulm, Germany) and a 53 μL saline chaser (to ensure that the entire furosemide dose entered the circulation), with MR data acquired for ~10 ± 2 min. Subsequently, the balanced electrolyte solution, Ringer's solution (B. Braun, Melsungen, Germany), was infused at a rate of 12 mL/kg for 10 min to replace fluid and electrolyte loss caused by furosemide, with MR data obtained for ~10 ± 2 min. Rats (*n* = 7) underwent interleaved *T*_2_* mapping and *T*_2_ mapping with short TE range before and after furosemide injection, and during the Ringer's solution infusion.

### 5.4 Spectral Analysis of the *T*_2_ Relaxation of Renal Parenchyma, and Tubular Fluid

To perform spectral analysis of the *T*_2_ relaxation in rat kidney, we acquired spatially resolved *T*_2_ decay curves acquired from rats (*n* = 2) during baseline with the long TE range protocol (Multi spin‐echo sequence, last echo time TE = 292.32 ms, Table 1) For spectral analysis, the data were then analyzed using a free fit with the nonnegative least squares (NNLS) algorithm implemented in the qMRLab module (https://github.com/qMRLab) for MATLAB (The MathWorks Inc., Natick, MA, USA) [61, 91, 92]. This was done to specify *T*_2_long_ and *T*_2_short_ range limits of renal tissue. The spectral analysis yields a spectrum of the contributions of all exponential basis vectors to the signal decay. The output is a *T*_2_ coefficient distribution with distinct log‐normal‐like peaks, where each peak corresponds to (a) major *T*_2_ compartment(s) [93, 94, 95]. The spectral analysis was performed without fixing *T*_2_ values or imposing a priori constraints on the number of peaks.

### 5.5 Decomposition of the *T*_2_ Decay to Obtain Tubular Volume Fraction

In healthy rats, parenchyma and blood compartments exhibit similar *T*_2_ relaxation times (9.4 T, *T*_2_ ≈52 ms in the medulla and a *T*_2_ ≈41 ms renal cortex, *T*_2_ ≈40 ms arterial blood), and only the tubular fluid has a considerably longer *T*_2_ relaxation (=150 ± 45 ms, 37°C, *n* = 2, pH = 6.0) [32, 33]. Based on this and on the outcome of our spectral analysis of the *T*_2_ decay, we used a bi‐exponential model of kidney tissue, in which the long *T*_2_ component is attributed to tubular fluid, while the short *T*_2_ components correspond to parenchymal tissue and blood compartments.

(1) $$I\left(t\right)={A_{1}}^{*}\exp\;\left(-t/T_{2\_\text{long}}\right)+{A_{2}}^{*}\exp\;\left(-t/T_{2\_\text{short}}\right)$$

*I* (*t*) is the signal amplitude and the evolution time used for *T*_2_‐weighting. *T*_2_long_ and *T*_2_short_ are the *T*_2_ relaxation times of the long and short components; *A*_1_ is the weight corresponding to the tubule water component, and A_*2*_ is the weight corresponding to renal parenchyma and blood.

The fitting was performed using nonlinear regression bi‐exponential fit of the *T*_2_ decay using MATLAB functions. This analysis was applied to *T*_2_ decay curves acquired from rats (*n* = 7) before and during furosemide intervention using the short TE range protocol (Table 1). For the fitting procedure, the limits for *T*_2_short_ were set to 10–40 ms, corresponding to parenchyma and blood components. *T*_2_long_ was fixed at 150 ms for tubular fluid. The short TE‐range protocol used for TVF assessment in interventional in vivo experiments was designed to balance the constraints of acquisition time, spatial resolution, and signal‐to‐noise ratio (SNR) [31], however, fixation is necessary since the maximum echo time (TE_max_ = 83.2 ms) is suboptimal to estimate *T*_2_long_ range up to 280 ms. Fixing some coefficients of the parameters is common practice to increase fit stability and to improve the sensitivity to physiological changes [96]. Mean absolute error (MAE) obtained from our phantom and simulation studies, using the same fixed *T*_2_long_ value, was sufficiently small, as previously demonstrated [31]. To compute the TVF, the ratio of the weight of longer *T*_2_ to the total sum of weights was determined.

(2) $$\mathrm{MAE}=\frac{\sum_{n=1}^{N}|x^{\prime }-x|}{N}$$

### 5.6 Validation of TVF Assessment in Phantom Study

To assess absolute TVF values acquired from the bi‐exponential decomposition with a fixed *T*_2_ from short TE range protocol, we developed a phantom consisting of two laboratory tubes (0.5 mL); one tube containing rat urine, and the other tube containing material mimicking *T*_2_/*T*_1_ relaxation properties of rat renal tissue. For the latter, pure water was doped with a mixture of MnCl_2_ and CuSO_4_ (Carl Roth GmbH, Karlsruhe, Germany) to mimic the *T*_2_ and T_1_ relaxation times characteristic of rat kidney tissue. Each tube was placed in a larger tube (2 mL) with distilled water, serving as a reference.

### 5.7 Simulation of TVF Estimation Error Arising From Fixation of *T*_2_long_

Our *T*_2_ measurements in urine provided *T*_2_ = 150 ± 45 ms. Because of this bandwidth of ±45 ms, we assessed potential errors of TVF estimation induced by fixing *T*_2_long_ to 150 ms. For this assessment, we used discrete *T*_2_ values of *T*_2_long_(min) = 100 ms and *T*_2_long_(max) = 200 ms for simulation. This is to demonstrate the impact of a wrong fixation of *T*_2_long_ = 150 ms on TVF measurements during the analysis of the short TE range protocol. We simulated the actual *T*_2_ echo modulation curve in a realistic Multi‐echo spin‐echo (MESE) MRI experiment, using the Bloch‐simulation toolkit (https://web.stanford.edu/~bah/software/epg/). The simulated *T*_2_ decay could be composed as:

(3) $$S\left(t\right)=M_{0}\sum_{j=1}^{2}\left(D_{j}\left(T_{2}\right).\mathrm{EPG}\left(T_{2},\theta^{-}\right)dT_{2}+\varepsilon (0,\mathrm{\sigma n})\right)$$

where *S*(*t*) is the signal amplitude; *M*_0_ represents the initial magnetization and signal intensity TE = 0, which is equal to the assumed proton density, and *j* = 2, the number of water compartments. *D*_*j*_ denotes the amplitude associated with each compartment, and *j* is the compartment index. EPG is the output of the extended phase graph algorithm; *θ* ^*−*^ stands for all other required parameters. The *ε*(0, σn) function indicates the additive white Gaussian noise. Gaussian distributed white noise was applied to the signal, such that SNR = mean(s)/*σ* is similar to the noise typically found in the magnitude images from in vivo studies. A synthetic dataset of simulated MESE *T*_2_ decay curves, resembling a two‐compartment model of kidney tissue, was generated by combining the signal amplitudes of the long component (tubular fluid; *T*_2_long_ = [100, 150, 200 ms]) with those of the short component (tissue/blood; *T*_2_short_ = 30 ms) across varying TVF values (ranging from 20% to 80% in 5% increments). The other parameters used in the simulations are similar to the short TE range protocol; TR = 500 ms, number of echoes = 13, first TE = 6.4 ms, inter‐echo time ΔTE = 6.4 ms, refocusing flip angle = 170° (to account for imperfect refocusing pulses), SNR = 100.

After the simulation, the data sets were fitted using a bi‐exponential model with the fixed *T*_2*_*long_ = 150 ms for all three ground truths of *T*_2*_*long_ = [100, 150 200 ms] to examine what the error of relative changes in TVF is if *T*_2*_*long_ is fixed to a value below, equal or higher than its true value. Relative changes refer to the difference in a TVF's value between two consecutive time points, expressed as a percentage of the value of the previous time point. It is calculated as:

(4) $$\mathrm{TVF}\;\text{Relative Change}=\frac{{\mathrm{TVF}}_{n}-{\mathrm{TVF}}_{n-1}}{{\mathrm{TVF}}_{n-1}}\times 100$$

where TVF_*n*_ is the value of TVF at time point *n*, TVF_*n*−1_ is the value of the TVF at the previous time point, *n*−1. To simulate image filtering broadly applied to MRI or averaging over ROIs, the results of nine simulations were averaged to form the final result. The Mean Absolute Error (MAE) as described in Eq. 2 quantifies the discrepancy between *x*′ (= the TVF relative changes observed from a fitting) and *x* (=the actual TVF relative changes), *x*′ is the estimated result of the *n* ^th^ trial, and N denotes the number of trials (*N* = 1000).

### 5.8 Image Analysis, Data Analysis and Statistics

TVF cartography was performed using a voxel‐wise bi‐exponential fit of the *T*_2_ decay with *T*_2_short_ constrained between 10 ms and 40 ms, and *T*_2_long_ fixed at 150 ms. Parametric maps of absolute *T*_2_* and *T*_2_ were calculated by pixel‐wise mono‐exponential fitting to the signal intensities of the *T*_2_*‐ and *T*_2_‐weighted images acquired at different echo times [63]. Median *T*_2_*, *T*_2_ and TVF values were calculated for regions‐of‐interest (ROI) placed in the renal cortex (CO), outer medulla (OM), and inner medulla (IM). ROI positioning was conducted using a standardized semiautomatic approach [97]. This procedure positions the ROIs (5 for CO and OM each, 3 for IM) such that they exclude the transition regions between renal layers to avoid partial volume effects.

For *T*_2_ mapping‐based determination of kidney size (KS), segmentation of the coronal mid‐slice cross‐sectional area of the kidney (here referred to as “kidney size,” KS) was performed using an automatic bean‐shaped model [63].

To separate *T*_2_* changes unrelated to deoxyhemoglobin from those associated with oxygenation (reflected in *T*_2_′), *T*_2_′ was estimated:

(5) $$\frac{1}{T_{2}^{*}}=\frac{1}{T_{2}}+\frac{1}{T_{2}^{\prime }}$$

Data were evaluated for Gaussian distribution using the Shapiro–Wilk test. Relative intervention‐mediated changes of *T*_2_, *T*_2_*, *T*_2_´, TVF, and kidney size were analyzed using the nonparametric repeated‐measures Friedman test, followed by Dunn's post hoc test for multiple comparisons. Correlations between relative changes in TVF, kidney size, *T*_2_*, *T*_2_ and *T*_2_´ were assessed using repeated‐measures correlation [64]. Data were analyzed using R v.3.6.3 with the packages “rstatix,” “dunn. test,” and “rmcorr” [98, 99, 100]. *p* < 0.05 was considered significant.

### 5.9 Near‐Infrared Spectroscopy Setup Used for the Assessment of Total Hemoglobin Concentration

In order to verify the postulated reduction in BVF after the furosemide bolus, we monitored the hemoglobin concentration per tissue volume in nine healthy rats with an in‐house built continuous‐wave NIRS setup [101]. Using fiber‐optic probes, we acquired the diffuse reflectance of light reemerging from kidney tissue at distances from the laser light source between 1 mm and 8 mm. The probe comprises two independent source fibers and seven detection fibers (Thorlabs Inc., FG200LCC, 200 μm core diameter, numerical aperture (NA) 0.27) with a spacing of 1 mm. The diffuse transmittance through the kidney was measured with three additional detection fibers (Thorlabs Inc., FT200UMT, 200 μm core diameter, NA 0.39) positioned at the opposite side of the kidney from the two sources. The light of nine lasers (Omicron‐Laserage GmbH, LightHub‐6 & LightHub‐4, 660, 685, 730, 785, 808, 850, 905, 980, and 1060 nm) was used as a source for 4.7 ms each in every acquisition cycle (50 ms). Using a 2 × 2 fiber switch (Leoni GmbH) this light was alternated between the two source fibers after every acquisition cycle. The light was detected with 10 avalanche photodiodes (Hamamatsu Photonics K.K.) equipped with reflective longpass filters, which reject all ambient light below 650 nm. Remaining background light was continuously monitored during the fiber switching time when all lasers were off and was subtracted from all signals.

A GPU‐based Monte Carlo simulation [102, 103] of the photon transport through the kidney was used to model the expected signals. The code was modified to produce histograms of the traveled path lengths of the photons in two layers (6 mm thick medulla and 2 mm thick renal cortex on both sides).

In order to reduce the number of open fit parameters, the wavelength dependence of the scattering coefficients was modeled using a power law function. The absorption was modeled as a linear combination of the spectra of oxy‐ and deoxy‐hemoglobin, water, fat, and cytochrome C [104, 105, 106]. Since the contribution of fat to the absorption is only very small, its concentration was fixed in the modeling to be half of the nonwater content. The optical properties were extracted from the measured data using a generalized least‐squares fit of the database lookup to the data.

The absolute sensitivities of all source‐wavelength‐detector combinations, their uncertainties and correlations were determined after every in vivo experiment by calibration measurements on 11 distinct epoxy‐resin‐ and PDMS‐based tissue phantoms with known absorption and scattering coefficients.

## Author Contributions

E.T.: conceptualization, data curation, formal analysis, investigation, methodology, software, validation, visualization, writing original draft. T.G.: conceptualization, data curation, formal analysis, investigation, methodology, software, validation, supervision, review, and editing. J.M.M.: data curation, formal analysis, software, validation, review, and editing. K.C: data curation, investigation, methodology, review, and editing. E.S: conceptualization, methodology, validation, review, and editing. T.N: conceptualization, funding acquisition, methodology, validation, resources, supervision, review and editing.

## Conflicts of Interest

The authors declare no conflicts of interest.

## Supporting information


**Figure S1:** apha70095‐sup‐0001‐FigureS1.docx.

## Data Availability

The data that support the findings of this study are available on request from the corresponding author. The data are not publicly available due to privacy or ethical restrictions.
