# Supplementary material for: Magnetic Resonance Cartography of Renal Tubule Volume Fraction During Diuretic Intervention
Source: Acta Physiol (Oxf). 2025 Aug 22;241(9):e70095. doi: 10.1111/apha.70095 (PMC12371858; doi:10.1111/apha.70095)
Supplement: Supplementary file 1 — Figure S1: apha70095‐sup‐0001‐FigureS1.docx. [file APHA-241-e70095-s001.docx]

# Supplementary Materials


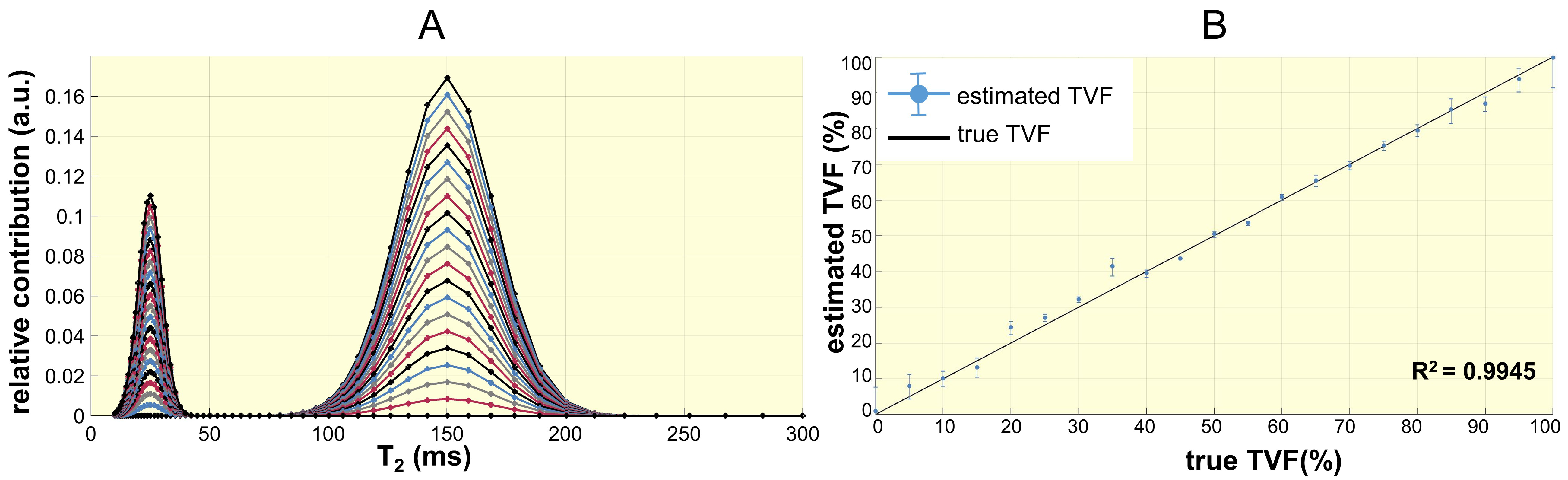


**Figure S.1**: **Assessing bi-exponential fitting error (A)** Simulated multi-compartment spectrum based on T_2_short_ and T_2_long_ values derived from spectral analysis. The multi-compartment model was developed for a multi-echo spin-echo signal decay using a Bloch simulation. Gaussian-distributed white noise was added to achieve an SNR (mean/σ) comparable to that observed in magnitude images from *in vivo* studies. Simulated T_2_ decay curves were generated by repeating simulations across 60 logarithmically spaced T_2_ values between 10 ms and 300 ms, with the long component's contribution varying from 0% to 100%. Other simulation parameters were consistent with those used in short TE range protocol (first TE=6.4 ms, ΔTE=6.4 ms, 13 echoes, α_refocusing pulse_°=170° to reflect imperfect refocusing). **(B)** TVF assessment with bi-exponential fitting, as similar to *in vivo* TVF assessment, was performed with T_2_short_ constrained to 10–40 ms and T_2_long_ fixed at 150 ms Results show strong agreement between ground truth TVF and fitted values.
